# Identification of Proteins Interacting with Cytoplasmic High-Mobility Group Box 1 during the Hepatocellular Response to Ischemia Reperfusion Injury

**DOI:** 10.3390/ijms18010167

**Published:** 2017-01-16

**Authors:** Tianjiao Zhang, Weiwei Wei, Olaf Dirsch, Thomas Krüger, Chunyi Kan, Chichi Xie, Olaf Kniemeyer, Haoshu Fang, Utz Settmacher, Uta Dahmen

**Affiliations:** 1 Experimental Transplantation Surgery, Department of General, Visceral and Vascular Surgery, Jena University Hospital, 07747 Jena, Germany; Tianjiaozhang@live.cn (T.Z.); wei.weiwei@med.uni-jena.de (W.W.); chuyi.kan@med.uni-jena.de (C.K.); popxcc@126.com (C.X.); Utz.Settmacher@med.uni-jena.de (U.S.); 2 Institute of Pathology, Klinikum Chemnitz gGmbH, 09116 Chemnitz, Germany; Olaf.Dirsch@gmail.com; 3 Department of Molecular and Applied Microbiology, Leibniz Institute for Natural Product Research and Infection Biology, Hans Knöll Institute, 07745 Jena, Germany; Thomas.Krüger@leibniz-hki.de (T.K.); olaf.kniemeyer@leibniz-hki.de (O.K.); 4 Department of Obstetrics and Gynecology, Wuhan Central Hospital, Wuhan 430014, China; 5 Department of Pathophysiology, Anhui Medical University, Hefei 230032, China; fang.haoshu@hotmail.com

**Keywords:** 2-dimensional electrophoresis (2DE), ischemic damage response, high-mobility group box 1 (HMGB1), ischemic injury, mass spectrometry (MS)

## Abstract

Ischemia/reperfusion injury (IRI) occurs inevitably in liver transplantations and frequently during major resections, and can lead to liver dysfunction as well as systemic disorders. High-mobility group box 1 (HMGB1) plays a pathogenic role in hepatic IRI. In the normal liver, HMGB1 is located in the nucleus of hepatocytes; after ischemia reperfusion, it translocates to the cytoplasm and it is further released to the extracellular space. Unlike the well-explored functions of nuclear and extracellular HMGB1, the role of cytoplasmic HMGB1 in hepatic IRI remains elusive. We hypothesized that cytoplasmic HMGB1 interacts with binding proteins involved in the hepatocellular response to IRI. In this study, binding proteins of cytoplasmic HMGB1 during hepatic IRI were identified. Liver tissues from rats with warm ischemia reperfusion (WI/R) injury and from normal rats were subjected to cytoplasmic protein extraction. Co-immunoprecipitation using these protein extracts was performed to enrich HMGB1-protein complexes. To separate and identify the immunoprecipitated proteins in eluates, 2-dimensional electrophoresis and subsequent mass spectrometry detection were performed. Two of the identified proteins were verified using Western blotting: betaine–homocysteine *S*-methyltransferase 1 (BHMT) and cystathionine γ-lyase (CTH). Therefore, our results revealed the binding of HMGB1 to BHMT and CTH in cytoplasm during hepatic WI/R. This finding may help to better understand the cellular response to IRI in the liver and to identify novel molecular targets for reducing ischemic injury.

## 1. Introduction

Ischemia/reperfusion injury (IRI) of the liver occurs inevitably in liver transplantation and after Pringle maneuver in major liver resections. Ischemia of the liver results in tissue hypoxia or anoxia. Reoxygenation caused by reperfusion aggravates hepatocellular injury by the subsequent inflammatory reaction.

IRI is associated with the expression of damage-associated molecular-pattern molecules (DAMPs). DAMPs include pathogen-associated molecular-pattern molecules (PAMPs) and endogenous danger signals (alarmins) [1]. Both PAMPs and endogenous danger signals can alert the host of danger, by initiating and promoting the innate and adaptive immune response, through their interaction with pattern recognition receptors [2]. Most endogenous DAMPs are nuclear or cytoplasmic proteins. When released to the extracellular space or exposed on the cellular surface following tissue injury or cellular stress, some play critical roles in orchestrating the response of cells to damage.

One important endogenous DAMP is high-mobility group box 1 (HMGB1). HMGB1 is a non-histone, nuclear protein and performs location-dependent roles. Nuclear HMGB1 stabilizes nucleosomes and facilitates gene transcription. During hepatic IRI, HMGB1 translates the damage into the subsequent inflammatory reaction. HMGB1 translocates from the nucleus to the cytoplasm and further to the extracellular space [3]. Extracellular HMGB1 interacts with receptor for advanced glycation end products (RAGE) and Toll-like receptors (TLR), mediating the release of proinflammatory cytokines as well as organ damage by activation of the c-Jun N-terminal kinase (JNK) and nuclear factor (NF)-κB pathways [4].

In contrast to the well-explored function of nuclear and extracellular HMGB1, little is known about the function of cytoplasmic HMGB1. Cytoplasmic HMGB1 may be involved in the cellular damage response to hepatic IRI, but the underlying mechanism is still unclear. Filling these knowledge gaps could help to better understand the role of HMGB1 in IRI.

**Scheme 1:**
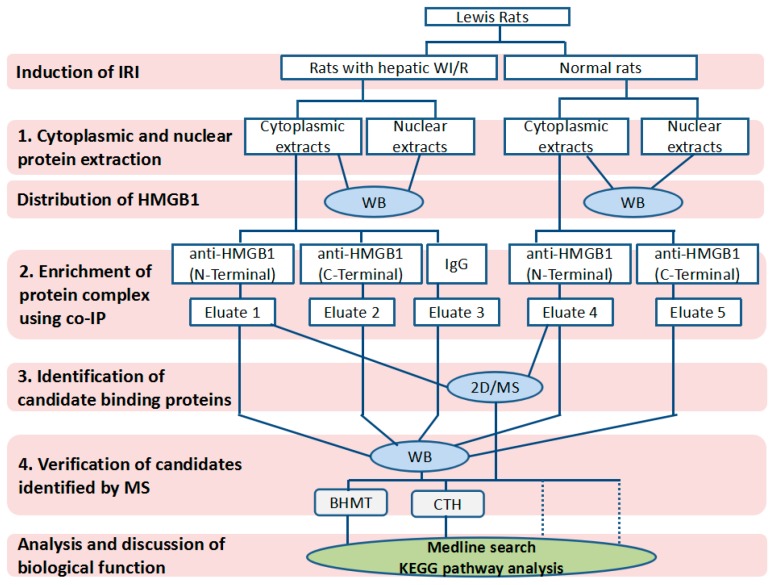
Study design. The steps marked with numbers 1–4 show the proteomic profiling process. The dotted lines represent the unverified candidate proteins. IRI: ischemia/reperfusion injury; WI/R: warm ischemia reperfusion; HMGB1: high-mobility group box 1; WB: western blotting; co-IP: co-immunoprecipitation; 2D/MS: 2-dimensional electrophoresis and mass spectrometry; BHMT: betaine–homocysteine *S*-methyltransferase 1; CTH: cystathionine γ-lyase; KEGG: Kyoto Encyclopedia of Genes and Genomes.

We hypothesized that cytoplasmic HMGB1 interacts with proteins involved in the hepatocellular damage response to IRI. To identify the proteins binding to cytoplasmic HMGB1 in hepatic warm ischemia reperfusion (WI/R) injury, we performed comparative proteomic profiling analysis of liver cytoplasmic extracts of rats with hepatic WI/R injury versus normal rats (see Scheme 1).

## 2. Results

### 2.1. Enrichment of Cytoplasmic High-Mobility Group Box 1 (HMGB1)-Binding Protein Complex from Warm Ischemia Reperfusion (WI/R) Liver Tissue

**Figure 1:**
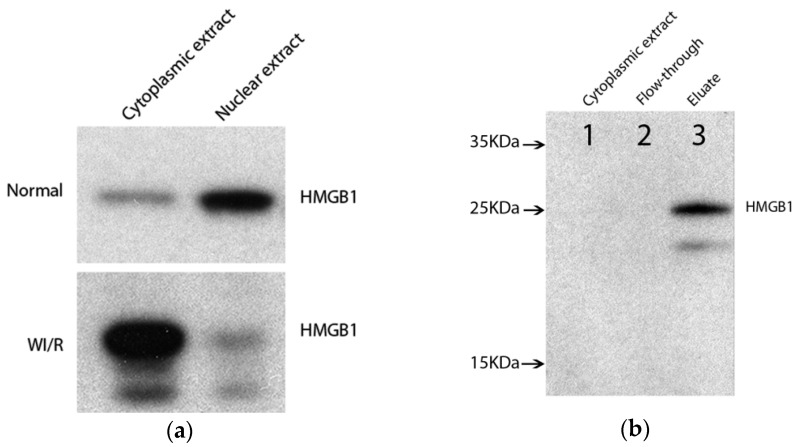
(**a**) Relative amount of high-mobility group box 1 (HMGB1) in cytoplasmic versus nuclear extract in warm ischemia reperfusion (WI/R) and normal liver. In normal rat liver tissue, HMGB1 level in nucleus was higher than in cytoplasm, whereas in WI/R liver tissue, HMGB1 protein was increased and higher in cytoplasm than in nucleus, suggesting translocation from nucleus into cytoplasm. Increased production as well as translocation from nucleus to cytoplasm implies that cytoplasmic HMGB1 is involved in hepatocellular response to damage caused by WI/R. Each lane contained the same protein amount (30 µg); (**b**) Enrichment of cytoplasmic HMGB1 protein from WI/R liver using co-immunoprecipitation (co-IP). HMGB1 was enriched into co-IP eluate. Considering the limited use of mild detergent in cytoplasmic protein extraction, this result allows the assumption that not only HMGB, but also the binding proteins, were enriched through the co-IP procedure. Each lane contained the same amount of protein (2 µg). We speculated that the faint band represents oxidized HMGB1, as shown previously by our group [5]. There was no signal detected in lane 1, for the loaded protein amount in cytoplasmic extract was too limited.

In the first step, we extracted nuclear and cytoplasmic proteins from normal liver and from livers subjected to 60 min ischemia and 6 h of reperfusion. We compared the relative amount of HMGB1 using Western blotting (WB). As expected, we detected more HMGB1 protein in the nuclear extracts from normal livers compared to the cytoplasmic protein extracts. Furthermore, we detected more HMGB1 in the cytoplasmic extract from the ischemic livers compared to the nuclear extracts (Figure 1a). This result was consistent with the immunohistochemical finding in our previous studies [3], confirming translocation of HMGB1 into the cytoplasm upon hepatic WI/R injury.

As a second step, we subjected cytoplasmic extracts from WI/R and normal liver tissues to co-immunoprecipitation (co-IP) for enrichment of cytoplasmic HMGB1-binding protein complexes. To confirm the enrichment of HMGB1 in co-IP eluates, cytoplasmic extracts, co-IP flow-through and eluates were subjected to WB. Compared with HMGB1 levels in the cytoplasmic extract and in the co-IP flow-through, HMGB1 protein in co-IP eluates was substantially increased (Figure 1b). We observed a faint band below the HMGB1 band (Figure 1a, lane 3). This band was also observed in the WB using protein extracts from WI/R liver but not from normal liver (Figure 1a).

### 2.2. Identification and Verification of Binding Proteins of Cytoplasmic HMGB1 in WI/R Liver Tissue

In the third step, the co-IP eluates were subjected to 2-dimensional electrophoresis (2DE) and mass spectrometry (MS), to separate and identify the proteins binding to cytoplasmic HMGB1 from WI/R. Spot patterns on the 2DE gels from both conditions were comparable. We chose the six regions with protein spots observed only on the 2DE gels from WI/R, but not presented in 2DE gels from normal tissue, for further MS analysis. Two regions contained rather small spots. These differential spots as well as 2 blank gel regions as controls were excised and subjected to MS/MS identification. Analysis of the two regions containing small spots did not result in detectable signals. Combining MS detection with image analysis of 2DE gels, analysis of the remaining four regions revealed five proteins reproducibly detected in two independent 2DE/MS experiments.

**Figure 2:**
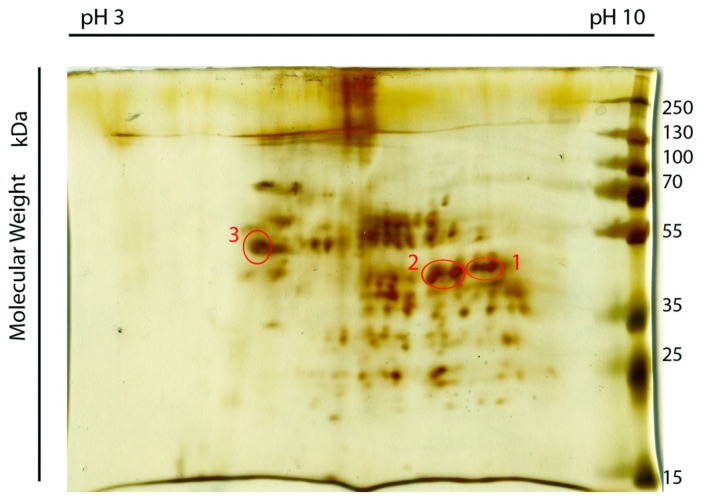
A representative 2-dimensional electrophoresis (2DE) gel. Co-IP eluate from WI/R cytoplasmic extract reacted with anti-HMGB1 was subjected to 2DE. Three unique protein regions (encircled in red) were identified through 2DE/MS identification and further verified using Western blot (WB).

Using the NCBI protein database, the present knowledge about the detected proteins was investigated to identify which proteins should be selected for the next verification study. For 1 out of the 5 proteins, identified as Chain A, Structural and Functional Importance of First-Shell Metal Ligands in the Binuclear Manganese Cluster of Arginase I (PubMed accession 33358012), neither relevant information nor appropriate reagents were available. Therefore, this protein was not subjected to further investigation. The remaining four proteins within three gel regions were subjected to the verification step and the pathway analysis. A representative 2DE gel image with the remaining three regions is shown in Figure 2. The functional information regarding the remaining four candidate proteins is shown in Table 1.

**Figure 3:**
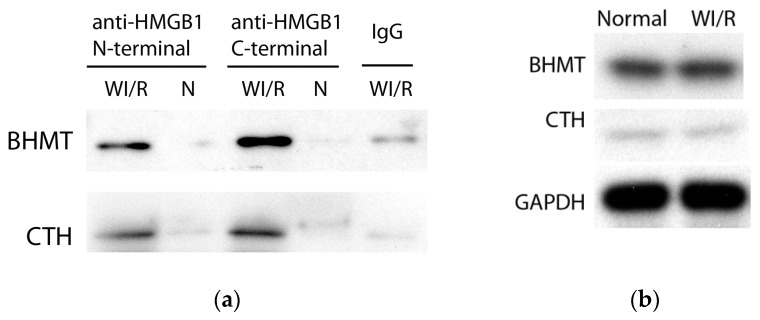
(**a**) Verification of identified proteins using Western blotting. The levels of target proteins were higher in co-IP eluates from WI/R cytoplasmic extract reacted with two different anti-HMGB1 antibodies. Extracts containing the same protein amount were subjected to reactions with different antibodies in co-IP experiments; the same volumes of eluates were subjected to WB in each lane within each candidate protein group. N, normal; WI/R, warm ischemia reperfusion; (**b**) Relative amount of the binding proteins in cytoplasm from WI/R versus normal livers. The relative amount of betaine–homocysteine *S*-methyltransferase 1 (BHMT) and cystathionine γ-lyase (CTH) in cytoplasm was not increased in WI/R animals.

As the fourth step, the additional co-IP and WB analysis thereafter were performed to verify the remaining four identified proteins. Our experiments revealed that 2 of 4 candidate proteins, betaine–homocysteine *S*-methyltransferase 1 (BHMT) and cystathionine γ-lyase (CTH), were to a larger extent immunoprecipitated by the bait protein HMGB1 from cytoplasmic extracts of WI/R tissue than from normal liver tissue. This finding was further verified in an additional control experiment. Using IgG as bait protein, neither candidate proteins were immunoprecipitated from the same WI/R cytoplasmic extracts. WB results are shown in Figure 3a. The co-IP plus immunoblotting analysis thus confirmed that BHMT and CTH are proteins co-precipitating with HMGB1 in cytoplasm during hepatic WI/R injury. Substantial evidence indicates that both binding proteins are involved in metabolic pathways, such as metabolism of methionine and cysteine (Table 1), implying their function in cellular response through these pathways. To investigate whether these two binding proteins were upregulated in WI/R animals, we further assessed their relative amount in cytoplasm from WI/R and normal livers. The results showed that neither of them was upregulated during WI/R (Figure 3b).

## 3. Discussion

This study aimed to identify putative binding proteins of cytoplasmic HMGB1 upon hepatic WI/R injury by use of comparative 2DE-MS proteomic analysis.

We separated cytoplasmic protein extracts to identify binding proteins of HMGB1 during hepatic IRI. The rat model with a reperfusion time of 6 h was selected based on previous experiments by our group revealing that, at this time point, cytoplasmic HMGB1 reached a peak [3]. Two different antibodies against HMGB1, one with a N-terminal and the other with a C-terminal immune epitope, were employed in this study. Using co-IP with anti-HMGB1 targeting the N-terminal, we enriched cytoplasmic HMGB1-binding protein complexes for 2DE and MS/MS identification. For verification, two different antibodies against HMGB1 as well as IgG were used to exclude nonspecific co-immunoprecipitated proteins. A similar study approach and experimental design has been proven successful in identification of protein–protein interaction by other groups [6,7,8]. The advantage of assessing endogenous binding proteins under various stimuli has been demonstrated by research involving both animals and plants [7,8].

In the 2DE gel, BHMT and CTH were identified in one region (Figure 2 region 2), probably because they have a similar molecular weight and charge (see Table 1). Also, BHMT was detected in two regions (Figure 2 region 1 and 2) suggesting the existence of at least 2 variants or modified forms. MS/MS identification suggested five candidates.

For Chain A, Structural and Functional Importance of First-Shell Metal Ligands in the Binuclear Manganese Cluster of Arginase I (PubMed accession 33358012), no information regarding the biological function was obtained upon a thorough Medline search. In addition, no reagents, especially no antibodies, were available for further verification studies. Therefore, this candidate was not subjected to verification in the current study, but could not be excluded as a binding protein of HMGB1. Following our experimental design, the remaining four candidates were subjected to verification and pathway analysis.

The candidate protein argininosuccinate synthase 1 (ASS1) was suggested by MS detection of a region 2 gel-piece. However, verification using either of the two anti-HMGB1 antibodies (the N-terminal anti-HMGB1 from preceding co-IP for MS and the C-terminal anti-HMGB1 antibody used for verification, respectively) resulted in comparable signals in IRI and normal liver tissues. Therefore, we speculate that the suggestion of ASS1 as candidate protein was merely a result of the MS-based algorithmic approach.

The third region was identified as ATP synthase β subunit (ATP5B), which could not be confirmed by the verification experiments using the additional anti-HMGB1 (C-terminal). This does not exclude ATP5B as binding protein of cytoplasmic HMGB1. The second antibody, targeting the C-terminal of HMGB1, could competitively influence the binding site of ATP5B in HMGB1, potentially leading to a false negative result in the confirmation experiment.

The binding proteins we identified are not the only partner proteins of cytoplasmic HMGB1 upon IRI. In some other studies, p53 and beclin1 were also demonstrated to interact with HMGB1 in cytoplasm [9,10]. However, in this study, 2DE analysis and MS identification did not detect these proteins as candidate binding proteins of cytoplasmic HMGB1. This could be the result of the low levels of these proteins, even below the sensitivity of the detection system we used.

**Scheme 2:**
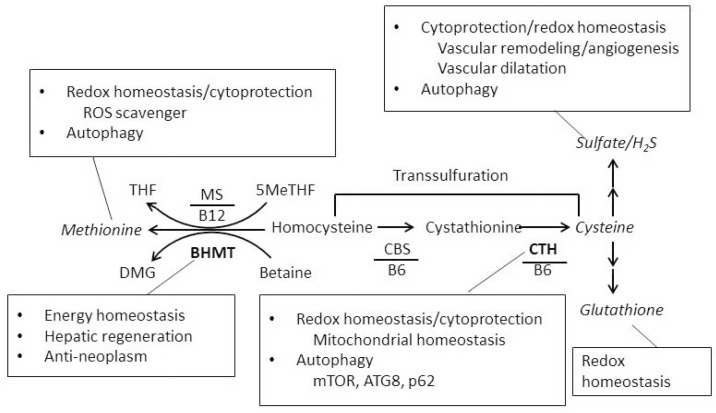
Functions of the binding proteins, as well as molecules downstream, in homocysteine metabolic pathways. THF, tetrahydrofolate; 5MeTHF, 5′ methyl tetrahydrofolate; MS, methionine synthase; DMG, dimethyl glycine; BHMT, betaine–homocysteine *S*-methyltransferase 1; CBS, cystathionine β-lyase; CTH, cystathionine γ-lyase; mTOR, the mammalian target of rapamycin; ROS, reactive oxygen species; ATG8, autophagy related protein 8.

Two proteins, BHMT and CTH, binding to cytoplasmic HMGB1 in hepatic WI/R, were identified in this study and further explored. Both proteins are enzymes involved in homocysteine metabolism [11,12]. BHMT catalyzes the synthesis of methionine from homocysteine and betaine. CTH catalyzes the second step in homocysteine transsulfuration pathway from cystathionine to cysteine. These metabolic pathways are involved in processes such as redox homeostasis and cytoprotection as well as autophagy (Scheme 2).

BHMT and CTH, as well as molecules further downstream, take part in maintaining the redox homeostasis, thereby contributing to cytoprotection, possibly also in hypoxia induced by IRI. Homocysteine metabolic pathways include molecules with antioxidative and cytoprotective properties [12,13], including methionine as well as cysteine, hydrogen sulfide, and glutathione. Methionine acts as a reactive oxygen species (ROS) scavenger in the oxidative stress response [13], protecting liver from oxidative damage induced by amino acid deprivation [14]. Cysteine is critical for generating hydrogen sulfide and glutathione [11]. Hydrogen sulfide is recognized as an endogenous signaling molecule and a cytoprotectant under oxidative stress [15,16]. Glutathione is an antioxidant molecule, found ubiquitously in the cell. The main function of glutathione is to maintain redox homeostasis in the cell to protect the cell from oxidative stress [16,17]. CTH knockout mice are highly susceptible to oxidative stress induced by mitochondrial toxins [18,19].

Interestingly, both BHMT and CTH are involved in autophagy pathways, implying that they may also modify hepatocellular autophagy during IRI. The two proteins are involved in the production of methionine and sulfide, respectively [20], which are negative regulators of the autophagy pathway. BHMT takes part in the generation of methionine. Methionine is sufficient to inhibit non-nitrogen-starvation (NNS)-induced autophagy in yeast strains [21]. CTH is responsible for the endogenous production of sulfide [22,23]. Metabolically generated sulfide in the cytoplasm of plant cells exerts a negative regulation on autophagy [24]. Deficiency of the l-cysteine desulfhydrase 1, the plant homologue of CTH, results in decreased hydrogen sulfide and, subsequently, in the accumulation of autophagy-related protein 8 (ATG8) [24]. In the myofibers of CTH-deficient mice, autophagy-related proteins LC3 as well as the specific autophagy substrate p62 were accumulated, indicating that CTH is required for autophagy in skeletal muscle [25]. CTH was decreased in fibroblasts from patients with Werner syndrome, leading to an excess activation of the mammalian target of rapamycin (mTOR) [23]. The activated mTOR suppressed autophagy, but was abrogated by administration of hydrogen sulfide. The ratio of LC3-II/LC3-I was regulated during treatment with hydrogen sulfide [23].

Recent publications suggest that cytoplasmic HMGB1 regulates autophagy. Cytoplasmic HMGB1 can bind to beclin1 and promote autophagy in some cell lines [9]. In mice with dextran sodium sulfate-induced colitis, lack of *Hmgb1* in intestinal epithelial cells resulted in exacerbation of inflammation which was attributed to a defect in autophagy. HMGB1 protects beclin1 and ATG5 from calpain-mediated cleavage during inflammation, allowing autophagy to proceed [26]. HMGB1 was also described as an autophagy-based alternative secretion substrate [27]. However, other studies revealed contradictory results regarding the role of HMGB1 in autophagy. Using conditional *Hmgb1* ablation in the liver, the in vivo study from the Schwabe laboratory showed that *Hmgb1* is dispensable for autophagy and mitochondrial function in adult mice [28].

During IRI, hypoxia induces oxidative stress, and simultaneously, oxidative stress aggravates the hypoxic condition in the tissue [29]. Homocysteine metabolism, in which BHMT and CTH play essential roles, affects the way cells respond to oxidative stress. In addition, both cytoplasmic HMGB1 and the identified binding proteins are involved in autophagy. These findings in our study suggest that cytoplasmic HMGB1 together with its interacting proteins may modulate the hepatocellular damage response by interfering with these processes.

Regarding the two identified proteins in this study, the molecular mechanism explaining how these putative partner proteins bind to cytoplasmic HMGB1 and what role this protein complex plays in hepatic IRI is not yet elucidated. We retrieved a few publications regarding the relevance of CTH in IRI, but none regarding the role of BHMT in IRI.

Some studies support the conventional view, that CTH as an essential enzyme in transsulfuration exerts a protective function against IRI. In CTH knockout mice, exacerbated myocardial and hepatic IRI were observed. This was due to increased oxidative stress and impaired endothelial NO synthesis [30]. The fasting-induced cardioprotection against IRI was absent in CTH^−/−^ mice. The protection provided by administration of hydrogen sulfide donor prior to IRI was suppressed as well. Quantitative analysis of reactive sulfur species indicated that CTH deficiency-induced excessive homocysteine diminished the protection of sulfide against IRI through capturing endogenous sulfide [31].

Intriguingly, a very recent study reported different results, where deficiency of CTH mitigated renal tubular damage caused by IRI. Interleukin 1-β, vascular cell adhesion molecule 1, tumor necrosis factor α, and intercellular adhesion molecule 1 were lower in IRI kidneys of CTH knockout mice. This indicated a loss of CTH-related decreased IRI in the kidney through reduction of inflammatory reactions. The author thus speculated that the reduced expression of CTH in kidney after IRI can be a cellular protective response [32]. In our current study, we observed no decrease in CTH after hepatic IRI (Figure 3b). However, we speculate that its binding to HMGB1 in hepatocytes may exert a similar function during cellular response to IRI.

Since there is substantial evidence that CTH is of relevance in IRI, our planned future study aims at further elucidating the underlying mechanism. We will explore how binding of CTH to cytoplasmic HMGB1 takes part in the hepatocellular response to IRI. Applying defined inhibitors for CTH in our future animal experiments, we will examine redox homeostasis and autophagy as well as the subsequent inflammation during hepatic IRI, and evaluate the effect of modulating these processes on the overall damage to the liver. With results of these experiments, we want to contribute to a better understanding of the biological relevance of the cytoplasmic HMGB1 protein complex in hepatocellular damage response.

## 4. Materials and Methods

### 4.1. Experimental Design

The experiments were designed to identify the proteins binding to cytoplasmic HMGB1 in WI/R liver tissues. Lewis rats subjected to 60 min hepatic ischemia and 6 h of reperfusion (*n* = 4) and normal animals (*n* = 3) were used. Nuclear and cytoplasmic proteins were separated.

Proteomic profiling consisted of 4 steps: (1) cytoplasmic protein extraction; (2) enrichment of cytoplasmic target proteins by co-IP; (3) separation and identification of target proteins using 2DE followed by MS; (4) verification of target proteins by co-IP and WB employing antibodies directed against candidate proteins detected by MS.

We regarded the enrichment of cytoplasmic target proteins by co-immunoprecipitation as the key step. Co-immunoprecipitation was performed using two distinct anti-HMGB1 antibodies, one targeting the C-terminal and the other targeting the N-terminal part of HMGB1. Furthermore, unspecific co-immunoprecipitation in WI/R cytoplasmic extracts was excluded using a control antibody (IgG). The study design is visualized in the schema in Scheme 1.

Tissue samples from seven animals were used in this study. Samples from three animals (two IRI and one normal) were used for 2DE. We performed two MS detection experiments based on two individual 2DE gels derived from two different co-IP runs. For the verification experiments, samples from four additional animals (two IRI rats and two normal rats) were used. All experiments were performed twice to confirm reproducibility of the results.

### 4.2. Animals and Selective Liver Ischemia

Male inbred Lewis rats were obtained from Charles River (Sulzfeld, Germany). The animals had a body weight of approximately 280–300 g. Animals were housed under standard animal care conditions and fed with rat chow ad libitum. Selective liver WI/R injury was induced by clamping the left lateral and median lobes for 60 min, followed by 6 h of reperfusion. At the end of this observation period, liver tissue from ischemic lobes was collected. Tissue from corresponding liver lobes from normal rats were used as control. All procedures were carried out according to the German Animal Welfare Legislation. Animal experiments were approved on 18 July 2013 by the Thüringer Landesamt für Verbraucherschutz, Thuringia, Germany (Approval-Number: 02-024/13).

### 4.3. Protein Extraction and Co-Immunoprecipitation

Nuclear and cytoplasmic protein extraction were performed after homogenizing the tissue using NE-PER Nuclear and Cytoplasmic Extraction Reagents (Thermo Scientific, Rockford, IL, USA). The cytoplasmic extraction reagent in this kit consists of a mild detergent (0.5% nonionic detergent), which does not interfere with protein–protein interactions, such as hydrogen bonds, ionic interactions, and van der Waals forces [33], making it suitable for IP applications. The protein extract was quantified using the Microplate BCA^TM^ protein assay kit (Thermo Scientific, Rockford, IL, USA). Halt^TM^ Protease and Phosphatase Inhibitor Cocktail (Thermo Scientific, Rockford, IL, USA) were added to the protein extract. Cytoplasmic protein extracts were subjected to co-IP (Pierce co-IP kit, Thermo Scientific, Rockford, IL, USA). An anti-HMGB1 antibody, targeting the N-terminal (Sigma-Aldrich, St. Louis, MO, USA) was used in co-IP in a ratio of 10 mg cytoplasmic protein extract to 10 µg antibody. Eluates were neutralized using 1 M Tris (pH 9.5). For the purpose of verification, the cytoplasmic protein extraction step was repeated using additional liver samples from two rats subjected to hepatic WI/R injury. Furthermore, the co-IP experiment was repeated using an additional anti-HMGB1 antibody, targeting the C-terminal (Sigma-Aldrich, St. Louis, MO, USA), and a control antibody IgG (Sigma-Aldrich, St. Louis, MO, USA). Three parallel co-IP experiments were performed for control purposes using (see Scheme 1): (1) rabbit IgG as control antibody reacting with cytoplasmic extract from WI/R liver tissue; (2) cytoplasmic extract from normal liver tissue as control lysate reacting with anti-HMGB1 (N-terminal); and (3) cytoplasmic extract from normal liver tissue as control lysate reacting with anti-HMGB1 (C-terminal).

### 4.4. Two-Dimensional Electrophoresis and Silver Staining

The Hoefer IEF100 isoelectric focusing unit (Hoefer, San Francisco, CA, USA) was used to perform the first-dimension electrophoresis. IPG Blue strips (7 cm, pH 3–10 and pH 5–8, linear), ampholytes (Servalyt^TM^, pH 3–10 and pH 5–8) and HPE IPG Overlay were purchased from Serva Electrophoresis (Heidelberg, Germany). Preparation of solutions and rehydration of strip gels were performed according to the IEF100 manual. Co-IP eluate (about 10 µg protein) was applied by cup loading after full denaturation with 2DE compatible buffer. The IEF100 preprogrammed protocol for 7 cm IPGs applying constant watt conditions was used. A two-step equilibration of IPG strips was performed before the second dimension SDS-PAGE. A minigel system (Hoefer, San Francisco, CA, USA) and 10% polyacrylamide gels were employed for SDS-PAGE. The IPG strip was placed on the top of the SDS gel. A piece of filter paper loaded with prestained peqGold Protein Marker V (Peqlab, Erlangen, Germany) was placed on the alkaline end of the IPG strip, and an overlay of hot agarose was used to seal the strip. SDS gels were transferred and subjected to silver staining. Pierce Silver Stain for Mass Spectrometry (Thermo Scientific, Rockford, IL, USA) was used according to the kit’s manual. Image acquisition of stained gels was performed using a scanner (Epson V750, Tokyo, Japan).

### 4.5. Mass Spectrometry Detection

Protein spots were excised from silver-stained gels and destained according to the silver stain kit manual. Gel pieces were washed using 50 mM NH_4_HCO_3_ and acetonitrile solutions, and digested with trypsin in 25 mM NH_4_HCO_3_ overnight at 37 °C according to the protocol of Shevchenko et al. 1996 [34]. Peptides were extracted from gel pieces and injected into an Ultimate 3000 nano RSLC system coupled to a QExactive Plus mass spectrometer (both Thermo Fisher Scientific). Peptides were analyzed as described earlier [35] with the exception of the following differences: Gradient elution with A (0.1% HCOOH) and B (0.1% HCOOH in 90% acetonitrile (ACN)) was as follows: 0–4 min at 4% B, 36 min at 35% B, 40 min at 50% B, 41–45 min at 96% B, 45.1–60 min at 4% B. The spray voltage of the Nanospray Flex Ion Source was set to 2.2 kV. Full scan precursor ion scan was performed within a mass range of *m*/*z* 300–1600 at 35 k full width at half-maximum (FWHM) resolution at a maximum injection time of 140 ms and an automatic gain control (AGC) target of 3e6. Top 10 precursor ions with an assigned charge state of *z* = 2–6 were selected for high-energy collisional dissociation (HCD) fragmentation at 30 V using nitrogen. Dynamic exclusion was set to 25 s. Using Proteome Discoverer 1.4, Thermo raw data were processed and searched against the NCBI database of *Rattus* using Mascot 2.4.1 (Matrix Science, London, UK). Further search parameters were exactly as described earlier [35]. The mass spectrometry proteomics data have been deposited to the ProteomeXchange Consortium via the PRIDE [36] partner repository with the dataset identifier PXD003538 and 10.6019/PXD003538.

### 4.6. Western Blotting

SDS-PAGE electrophoresis was performed using a minigel system (Hoefer, San Francisco, CA, USA) and 12% acrylamide gels. Transfer of proteins to PVDF membranes (GE Healthcare, Braunschweig, Germany) was performed employing a tank transfer unit (Hoefer, San Francisco, CA, USA). Membranes were blocked using 10% milk solution (10% nonfat milk powder and 0.1% Tween 20 in TBS solution). For HMGB1 detection, different types of samples containing the same protein amount were loaded in each lane. Anti-HMGB1 antibodies were purchased from Sigma (Sigma-Aldrich, St. Louis, MO, USA). For verification, the same volumes of co-IP eluates were loaded in each lane. The antibodies specific for the candidate proteins identified by 2DE/MS were used separately. Anti-ATP5B and anti-CTH were purchased from Sigma (Sigma Life Science, Stockholm, Sweden and Sigma-Aldrich, Wuxi, China). Anti-BHMT was purchased from Santa Cruz Biotechnology (Santa Cruz, CA, USA). Membranes were probed with secondary goat-anti-rabbit and rabbit-anti-goat antibodies (Abcam, Cambridge, UK) conjugated to horseradish peroxidase. Signals were detected using Lumi-Light Western Blotting Substrate (GE Healthcare, Buckinghamshire, UK) and were exposed to high sensitivity films (GE Healthcare, Tokyo, Janpan) for autoradiography. Digitalization of films was performed using a film scanner (Epson V750, Tokyo, Japan). Information regarding the biological function of the verified proteins was retrieved applying PubMed and KEGG pathway analysis.

## 5. Conclusions

Using a comparative proteomic analysis, we identified two proteins binding to cytoplasmic HMGB1 during hepatic WI/R injury. Both binding proteins are involved in homocysteine metabolic pathways, which are of importance for redox homeostasis and autophagy. These processes are relevant for the hepatocellular response to IRI. Our results hereby suggest that cytoplasmic HMGB1 interacting with binding proteins may be involved in regulating the hepatocellular response to IRI by interfering with these processes. This may lead to identification of novel molecular targets to reduce damage and allow improved restoration of cellular function.

## Figures and Tables

**Table 1:** Candidate proteins by MS identification and image analysis.

Accession ^1^	Protein	Region No.	Score	Coverage	No. of Peptides	MW(kDa) ^2^	calc. pI ^3^	Biological Function ^4^
13540663	Betaine—homocysteine *S*-methyltransferase 1	1	566.87	10.57	6	44.9	7.91	Metabolic pathways; Cysteine and methionine metabolism
13540663	Betaine—homocysteine *S*-methyltransferase 1	2	566.74	16.46	10	44.9	7.91	
56030	Cystathionine γ-lyase	2	80.63	10.44	4	39.7	8.03	Metabolic pathways; Cysteine and methionine metabolism; Sulfide production
25453414	Argininosuccinate synthase	2	291.93	17.23	10	46.5	7.78	Alanine, aspartate and glutamate metabolism; Arginine biosynthesis; Biosynthesis of amino acids; Urea cycle
1374715	ATP synthase β subunit	3	360.79	8.42	4	51.2	5.02	Oxidative phosphorylation; Metabolic pathways

^1^ Accession number in protein database of NCBI; ^2^ Molecular weight; ^3^ Calculated isoelectric point; ^4^ Based on Medline search and KEGG pathway analysis.

## Abbreviations

IRI	Ischemia reperfusion injury
WI/R	Warm ischemia reperfusion
WB	Western blotting
co-IP	Co-immunoprecipitation
2DE/MS	2-dimensional electrophoresis and mass spectrometry
BHMT	Betaine–homocysteine *S*-methyltransferase 1
CTH	Cystathionine γ-lyase
